# A wearable real‐time particulate monitor demonstrates that soaking hay reduces dust exposure

**DOI:** 10.1111/evj.14425

**Published:** 2024-10-27

**Authors:** Kathleen M. Ivester, Ji‐Qin Ni, Laurent L. Couetil, Thomas M. Peters, Marcus Tatum, Lynn Willems, Jae Hong Park

**Affiliations:** ^1^ College of Veterinary Medicine Purdue University West Lafayette Indiana USA; ^2^ Department of Agricultural and Biological Engineering Purdue University West Lafayette Indiana USA; ^3^ Department of Occupational and Environmental Health University of Iowa Iowa City Iowa USA; ^4^ School of Health Sciences Purdue University West Lafayette Indiana USA

**Keywords:** asthma, calibration, horse, optical sensor, organic

## Abstract

**Background:**

Affordable particulate matter (PM) monitors suitable for use on horses will facilitate the evaluation of PM mitigation methods and improve the management of equine asthma.

**Objective:**

Calibrate a real‐time wearable PM monitor (Black Beauty [BB]) and compare the PM exposures of horses fed dry or soaked hay.

**Study design:**

Laboratory calibration; complete cross‐over feed trial.

**Methods:**

Side‐by‐side sampling with BB monitors and tapered element oscillating microbalances (TEOMs) was performed under varying concentrations of PM from alfalfa hay. Linear regression was used to derive a calibration formula for each unit based on TEOM PM measurements. Precision was evaluated by calculating the coefficient of variation and pairwise correlation coefficients between three BB monitors. PM exposure was measured at the breathing zone of 10 horses for 8 h after they were fed dry or soaked hay. Repeated measures generalised linear models were constructed to determine the effect of hay treatment and measurement duration (initial 20‐min vs. 8‐h) upon exposure to PM with diameters smaller than or equal to 10 μm (PM_10_) and 2.5 μm (PM_2.5_).

**Results:**

BB monitor PM_2.5_ and PM_10_ measurements were linearly correlated with TEOM data (coefficient of determination *r* ^2^ > 0.85 and *r* ^2^ > 0.90 respectively), but underestimated PM_2.5_ mass concentrations by a factor of 4 and PM_10_ concentrations by a factor of 44. Measures from the three BB monitors had a coefficient of variation <15% and pairwise *r* > 0.98. Feeding soaked hay significantly reduced average PM_2.5_ exposures (20‐min: dry: 160 μg/m^3^, soaked: 53 μg/m^3^, *p* < 0.0001; 8‐h: dry: 76 μg/m^3^, soaked: 31 μg/m^3^, *p* = 0.0008) and PM10 exposures (20‐min: dry: 2829 μg/m^3^, soaked: 970 μg/m^3^, *p* < 0.0001; 8‐h: dry: 1581 μg/m^3^, soaked: 488 μg/m^3^, *p* = 0.008).

**Main limitations:**

No health outcome measures were collected.

**Conclusions:**

With appropriate corrections, the BB monitor can be used to estimate horse PM exposure. While 20‐min measurements yielded higher estimates of exposure than 8‐h measurements, both intervals demonstrate that soaking hay reduces PM exposures by more than 50%.

## 1 INTRODUCTION

Exposure to airborne particulate matter (PM) is central to the development and exacerbation of asthma in horses. Horses develop airway inflammation, obstruction, and remodelling in response to increased particulate exposures.^1^, ^2^, ^3^ Though defined to assess the risk of human exposure, both respirable PM (nominally particles with diameters 4 μm) and PM_10_ (PM with diameters 10 μm) particle size fractions have been found pertinent to respiratory health in horses.^4^, ^5^, ^6^, ^7^, ^8^ When confined to stalls, horses experience increased PM exposures,^9^ and the magnitude of this increase appears to be largely determined by the type of forage^10^, ^11^ and the method^6^ of feeding. Most stabled horses are fed hay, which is a rich and complex polydisperse source of organic PM.^12^ While there are many methods to decrease PM exposure in stabled horses, soaking hay is simple and practical with minimal additional costs. Though multiple studies have examined changes in the hygiene of soaked hay over time,^13^, ^14^, ^15^ PM exposure of horses fed soaked hay is incompletely characterised. Reduced respirable dust in the breathing zone of a single horse was demonstrated when soaked hay was fed,^16^ neglecting the variability due to differences in individual horse behaviour, an important determinant of exposure.^17^

Investigations into the health effects of PM inhalation have been hampered by the expense and labour associated with accurate measures of personal exposure in epidemiological studies involving horses and humans alike. Technological advances have enabled the development and commercialisation of low‐cost, compact, real‐time PM monitors that have the potential to greatly facilitate personal exposure assessments. However, the performance of these low‐cost monitors can vary between units^18^, ^19^, ^20^, ^21^ as well as with the composition, concentration, and size distribution of the aerosol,^22^, ^23^, ^24^, ^25^, ^26^ necessitating the evaluation and calibration of low‐cost PM monitors using reference grade instruments under conditions pertinent to the planned exposure measurements. Furthermore, because the lower limit of detection does not depend upon duration of sampling, real‐time PM monitors can provide data over very short periods of time. Little information exists to determine duration necessary to provide an accurate representation of exposure. Published horse PM exposure studies have included sampling times ranging from 60 s^7^ up to 6 h.^8^

Therefore, this study introduces a purpose‐built, wearable PM monitor designed for use on horses, details its calibration under varying concentrations of hay dust, and demonstrates its use in comparing PM exposures when horses are fed dry hay to when they are fed soaked hay using short (20‐min) and extended (8‐h) measurements.

## 2 MATERIALS AND METHODS

### 2.1 PM monitors

**FIGURE 1:**
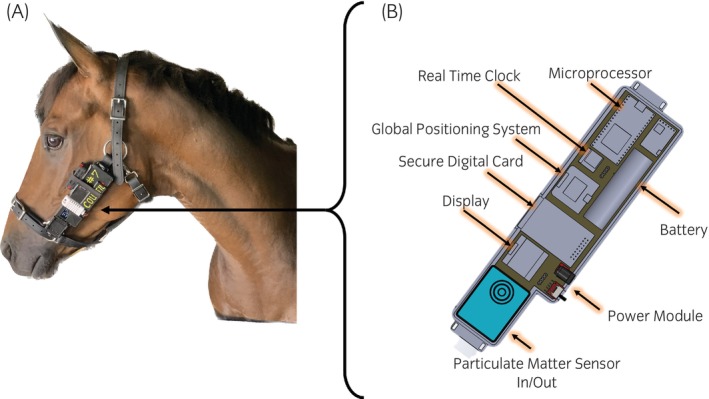
Black Beauty (BB) PM monitor. Shown mounted on the halter of a horse (A) and a labelled schematic without the top of the case (B).

Three Black Beauty (BB) monitors were tested. The BB monitor (Figure 1) is a custom‐built, ruggedised PM monitor that incorporates an optical PM sensor (PMS7003, Plantower Technology), temperature, and relative humidity sensors (AM2302, Aosong), and a global positioning system (GPS) receiver (GT‐U7, Goouu Tech). The PMS7003 sensor provides mass concentrations for PM with diameters ≤1 μm (PM_1_), 2.5 μm (PM_2.5_), and 10 μm (PM_10_) in μg/m^3^, and particle number concentrations (particles/0.1 L) in six size bins: >0.3 μm, >0.5 μm, >1.0 μm, >2.5 μm, >5.0 μm, and >10 μm. A custom‐designed printed circuit board connects the sensors and supporting electronics. A microprocessor (Teensy 3.2) samples all the sensors on 5‐s intervals and logs time‐stamped data along with GPS coordinates on a secure digital (SD) card. A liquid crystal display is added to provide real‐time sensor readings and diagnostic information.

The case mounts on a standard halter to allow on‐horse measurements near the breathing zone. The system was designed to be light with rounded edges to prevent injury to the horse.

The Tapered Element Oscillating Microbalance (TEOM) Ambient Particulate Monitor Model 1400a (Thermo Fisher Scientific) is an Environmental Protection Agency (EPA)‐designated automated equivalent method for PM_10_ and PM_2.5_.^27^ One TEOM equipped with a PM_10_ sampling inlet and another equipped with a PM_2.5_ inlet were used as the reference instruments and configured as previously described.^28^

Because the response time of the TEOM differs significantly from small optical sensors, six DC1700‐PM PM_2.5_/PM_10_ Air Quality Monitors (Dylos monitors, Dylos Corporation) were used to quantify the response time of the TEOM units (Data S1).

### 2.2 PM generation

Conventionally produced, good‐quality alfalfa hay was used as the PM source. Dust was released from the hay using a custom‐built agitator consisting of an open‐faced cylindrical chamber (diameter of 30.5 cm) and a central metal shaft (diameter of 2.5 cm) with six orthogonal rods (diameter of 0.95 cm; thread count 18) (Figure S1). A variable speed motor turned the shaft at speeds from 0 to 670 revolutions per minute. The test PM was generated at various quasi‐steady state concentrations by varying the amount of hay (50–250 g) and agitation speed.

### 2.3 Test chamber and experimental setup

**FIGURE 2:**
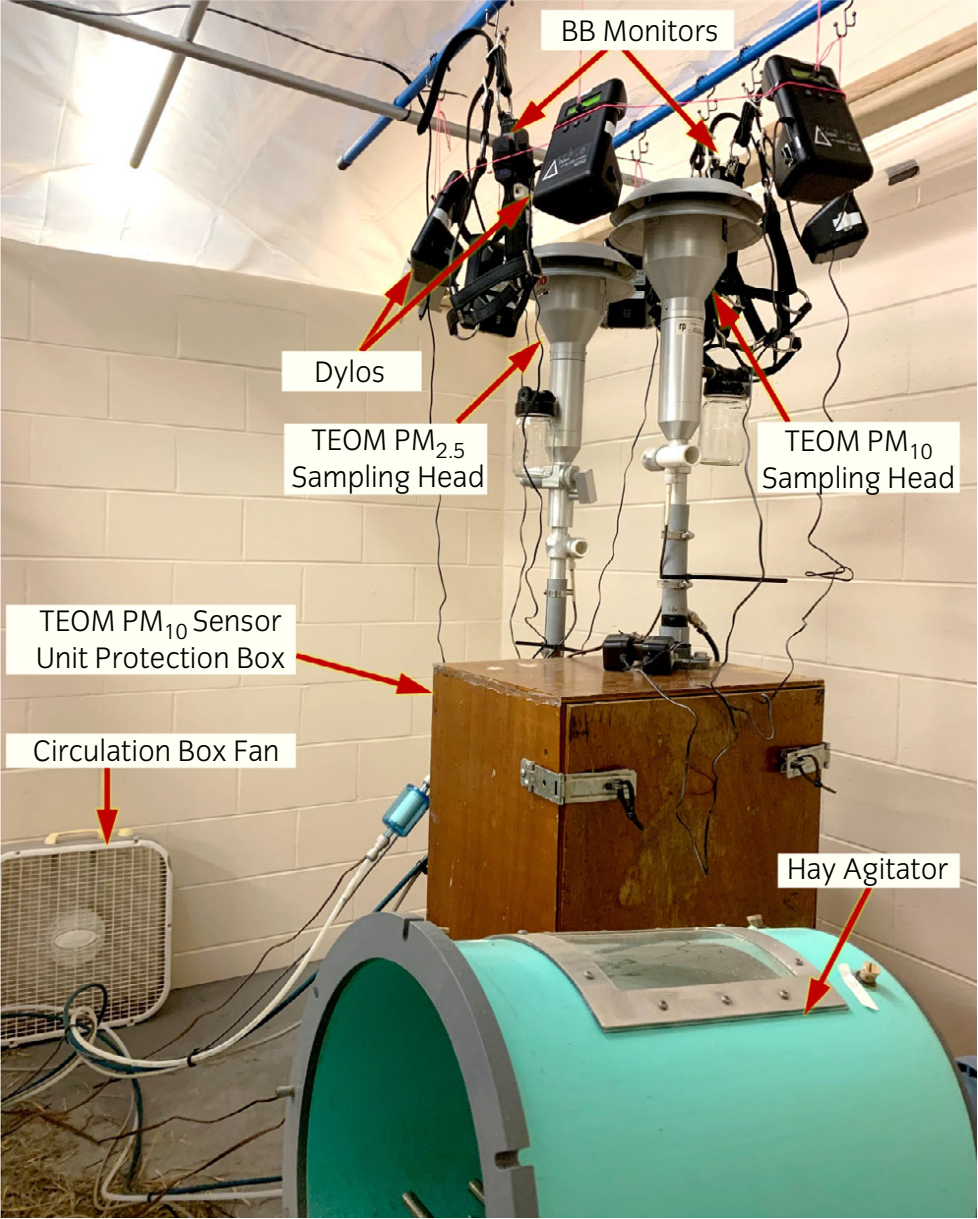
Experimental setup with co‐located PM monitors, hay agitator, and box fan in the test chamber.

The test chamber was located within a cement‐block horse stall in a climate‐controlled barn at the Purdue University College of Veterinary Medicine Equine Sports Medicine Center (Figure 2 and Data S2).

Quasi‐steady state conditions were created for 5‐min intervals over a range of PM concentrations. These intervals were identified from 1‐min averaged TEOM data with a coefficient of variation (CV) <1% over the course of at least 5 min. The corresponding interval was identified in the Dylos logged data. TEOM data recorded at 1‐min intervals was analysed side by side with the 1‐min interval data of the Dylos units to calculate the response time of the TEOM units. From the time stamp of the Dylos data, the corresponding 5‐min interval was identified in the data from three BB monitors.

As a measure of BB monitor precision, the CV was calculated for PM_2.5_ and PM_10_.^19^ In addition, Pearson correlation coefficients were calculated for each pairwise comparison between the BB monitors.

The 5‐min averaged TEOM and BB data from identified intervals were plotted to judge the linearity of the BB response to PM. Univariate linear regression with TEOM data as the dependent variable and BB data as the predictor variable was performed to calculate the coefficient of determination (*r* ^2^) and root mean square error (RMSE in μg/m^3^) between the two methods as well as to derive a calibration formula for each BB unit.^29^ Graphical methods were used to confirm that the model assumptions were met. In addition, normality of model residuals was tested using the Shapiro–Wilk test.

### 2.4 Cross‐over feed trial

A convenience sample of 10 horses of various breeds with median (range) age of 21 (6–23) years and median (range) weight of 542 (482–605) kg from the Purdue University teaching herd was used. Horses remained clinically healthy during the study. All horses had been previously acclimatised to wearing the BB monitors. Horses were housed in box stalls (3.7 × 3.7 m^2^) within an enclosed, climate‐controlled barn. Good quality grass hay from a single source was fed at a rate of 4 flakes (5–7 kg) twice daily throughout the course of the study. In a cross‐over design, PM exposure was measured at the breathing zone using BB monitors while horses were fed either dry or soaked hay. Each day of testing, BB monitors were secured to break‐away halters and placed on the horses (Figure 1) in the morning after stall cleaning but before feeding. Horses were then provided with either dry hay or hay that had been placed in a large tub of water and completely submerged for 15 min immediately before feeding. Hay was submerged for 15 min to ensure complete saturation without changing the nutritional content of the hay. The monitors remained on the horses and recorded PM_2.5_ and PM_10_ every 5 s for 8 h. Each horse underwent exposure assessment on 2 days between 25 February and 26 April, and was fed dry hay on the first occasion and soaked hay on the second (Figure S2). Horses were fed from the ground with identical placing of the dry and soaked hay.

Raw data collected from the BB monitors were corrected according to the calibration formula and average PM_2.5_ and average PM_10_ exposures were calculated for two time intervals: (1) the initial 20 min of measurement after the forage was placed in the stall (short) and (2) the entire 8 h measurement period (extended). To determine the effect of hay treatment upon PM measures, separate generalised linear repeated measures models^30^ were constructed using short and extended measures of average PM_2.5_ and PM_10_ exposure. To determine the effect of measurement duration on PM measures, generalised linear repeated measure models were constructed including the effects of hay treatment, measurement duration, and the interaction between hay treatment and measurement duration. All models were constructed under the assumption of lognormal distribution with statistical significance set at *p* < 0.05. Tukey‐adjusted *p*‐values were to control type I error in the case of multiple pair‐wise comparisons.

All statistical analyses were performed using SAS (Statistical Analysis Software v9.4, SAS Institute Inc., Cary, NC).

## 3 RESULTS

### 3.1 BB monitor precision

**FIGURE 3:**
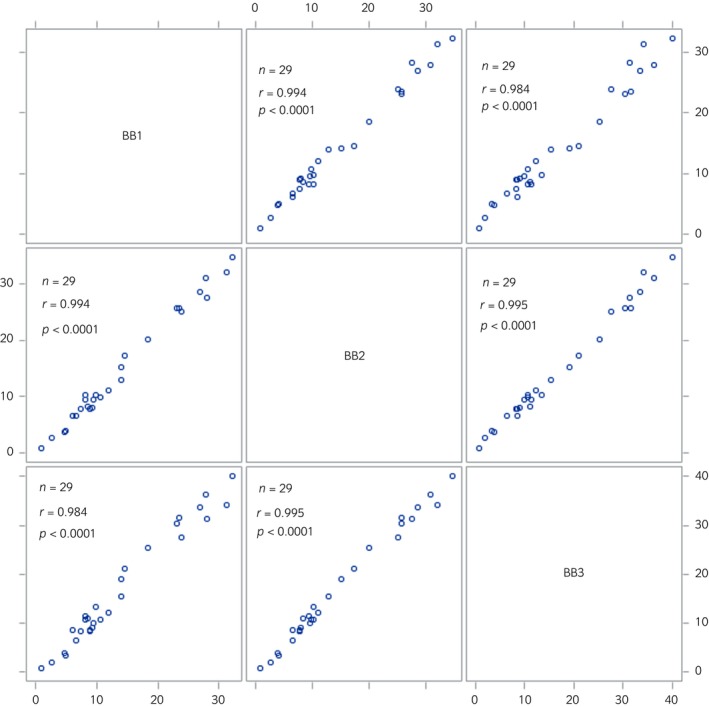
Scatter plot correlation matrix for BB monitor PM_2.5_ measures (μg/m^3^). BB, Black Beauty particulate matter monitor; PM_2.5_, particulate matter with an aerodynamic diameter ≤2.5 μm.

**FIGURE 4:**
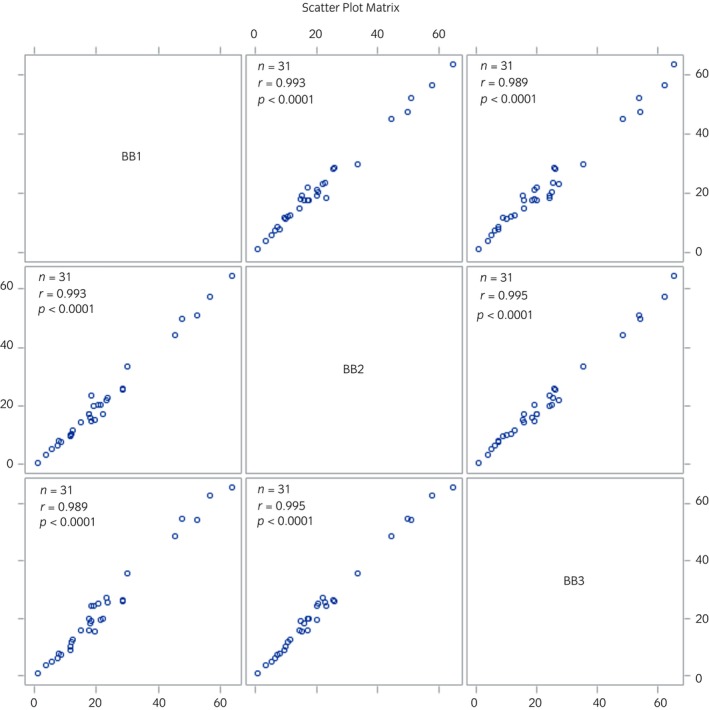
Scatter plot correlation matrix for BB monitor PM_10_ measures (μg m^−3^). BB, Black Beauty particulate matter monitor; PM_10_, particulate matter with an aerodynamic diameter ≤10 μm.

The CV between BB monitor units was 14.37% for PM_2.5_ measurements and 12.79% for PM_10_ measurements, indicating acceptable agreement between units.^31^ Furthermore, the correlation between units was very strong (*r* > 0.98, Figures 3 and 4).

### 3.2 BB monitor bias

The TEOM data recorded at 1‐min intervals were analysed side by side with the 1‐min interval data of the Dylos units, quantifying the lag in the response of the TEOM units as 4 min. Quasi‐steady state PM_2.5_ concentrations recorded by the TEOM ranged from 3.5 to 163.3 μg/m^3^, and PM_10_ concentrations ranged from 9.7 to 2543 μg/m^3^, with a total of 31 PM_10_ and 29 PM_2.5_ data segments identified that met the requirement of CV <1% over the course of the identified 5‐min interval. Regression analysis revealed a strong linear relationship between the BB monitor PM measurements and the reference gravimetric measurements provided by the TEOM for both PM_2.5_ (Table 1) and PM_10_ (Table 2), with *r* ^2^ values ranging from 0.86 to 0.94. However, BB monitors had significant bias and underestimated PM concentrations for both PM_2.5_ and PM_10_ as evidenced by the slopes of the regression equations, which ranged from 3.6 to 4.48 and 42 to 45.84, respectively (Tables 1 and 2). Given the high degree of correlation between BB units, measures from the three BB units were then averaged to derive a universal calibration formula for future use regardless of BB unit (Tables 1 and 2; Figures S3 and S4).

**TABLE 1:** Results of linear regression models comparing BB monitor reported PM_2.5_ to TEOM PM_2.5_ in 29 observations (*p*‐values <0.0001 for all).

Monitor	*r* ^2^	RMSE (μg/m^3^)	Regression equation
BB1	0.864	16.5	TEOM=4.48×BB1PM2.5−5.17
BB2	0.889	14.9	TEOM=4.16×BB2PM2.5−2.82
BB3	0.919	12.7	TEOM=3.60×BB3PM2.5−2.58
Averaged BB	0.898	14.3	TEOM=4.07×BBPM2.5−3.82

Abbreviations: BB, Black Beauty monitor; PM_2.5_, particulate matter with an aerodynamic diameter ≤2.5 μm; *r* ^2^, coefficient of determination; RMSE, root mean square error; TEOM, tapered element oscillating microbalance.

**TABLE 2:** Results of linear regression models comparing BB monitor reported PM_10_ to TEOM PM_10_ in 31 observations (*p*‐values <0.0001 for all).

Monitor	*r* ^2^	RMSE (μg/m^3^)	Regression equation
BB1	0.912	226.8	TEOM=45.8×BB1PM10−113.7
BB2	0.923	211.5	TEOM=44.6×BB2PM10−51.6
BB3	0.937	192.1	TEOM=42.0×BB3PM10−65.3
Averaged BB	0.930	202.8	TEOM=44.3×BBPM10−81.5

Abbreviations: BB, Black Beauty monitor; PM_10_, particulate matter with an aerodynamic diameter ≤10 μm; *r* ^2^, coefficient of determination; RMSE, root mean square error; TEOM, tapered element oscillating microbalance.

### 3.3 Effect of soaking hay and measurement duration on PM exposures

**FIGURE 5:**
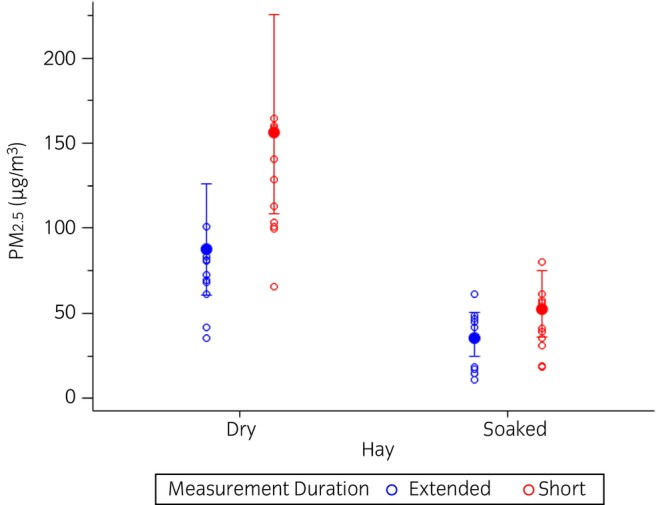
Generalised linear model of PM_2.5_ exposures when horses were fed dry or soaked hay according to short or extended measurements. Data are displayed on the original scale. Solid circle = model least square group mean; whiskers = 95% confidence interval for the mean; open circles = individual data points. Short, exposure measures obtained for the first 20 min after forage was supplied; Extended, exposure measures obtained over the course of 8 h after forage was supplied; PM_2.5_, particulate matter with an aerodynamic diameter ≤2.5 μm.

**FIGURE 6:**
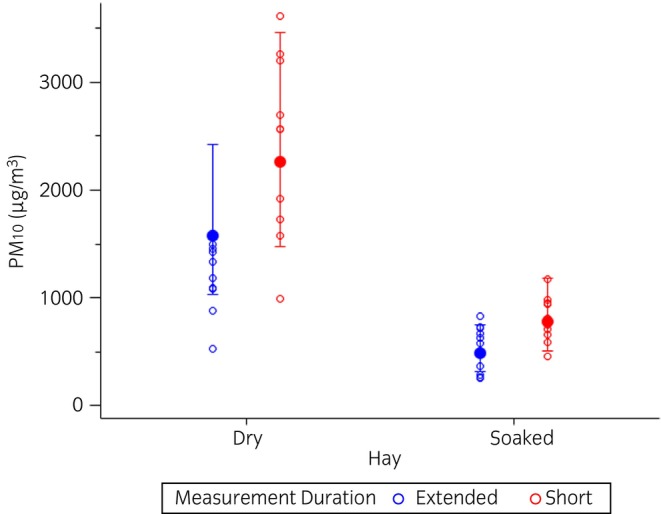
Generalised linear model of PM_10_ exposures when horses were fed dry or soaked hay according to short or extended measurements. Data are displayed on the original scale. Solid circle = model least square group mean; whiskers = 95% confidence interval for the mean; open circles = individual data points. Extended, exposure measures obtained over the course of 8 h after forage was supplied; PM_10_, particulate matter with an aerodynamic diameter ≤10 μm; Short, exposure measures obtained for the first 20 min after forage was supplied.

All horses completed the study, tolerating exposure assessment and change in forage from dry hay to soaked hay with no discernable change in appetite or behaviour. Both short and extended measurements demonstrated that feeding soaked hay resulted in a significant reduction in both mean PM_2.5_ exposures (*p* < 0.0001 and *p* = 0.0008, respectively) and mean PM_10_ exposures (*p* < 0.0001 and *p* = 0.008, respectively) (Figures 5 and 6; Table S1).

Models including the effects of hay treatment, measurement duration, and the interaction between hay treatment and measurement duration upon PM exposure indicated that the short measurement duration yielded a significantly higher estimate of PM_2.5_ exposures (*p* = 0.001), but not PM_10_ exposures (*p* = 0.06). However, there was no interaction between hay treatment and measurement duration (PM_2.5_: *p* = 0.5; PM_10_: *p* = 0.8, Figures 5 and 6).

## 4 DISCUSSION

Following its calibration using an EPA‐designated automated equivalent method gravimetric instrument over a wide range of hay‐derived organic dust concentrations, the custom‐built BB PM monitor demonstrated that soaking hay reduces PM exposures as measured at the breathing zone of horses.

The BB monitors displayed a strong correlation with the reference TEOM instruments for both PM_2.5_ (*r* ^2^ > 0.86) and PM_10_ (*r* ^2^ > 0.91). However, the BB monitors significantly underestimated PM_2.5_ and PM_10_ mass concentrations, as evidenced by the slopes obtained in the regression models. This finding contrasts with comparisons made between the PMS7003 sensor and reference instruments in the measurement of ambient particulate air pollution, where these low‐cost sensors tended to over‐estimate PM mass concentrations.^24^, ^29^, ^32^ However, the AirBeam2, a commercially available PM monitor incorporating the PMS7003 sensor, significantly underestimated PM_2.5_ concentrations of an oil‐based aerosol reagent.^33^ Taken with our data, these findings serve to emphasise the importance of calibration with a PM source that is pertinent to the intended use of the sensor.

Agreement among the three BB monitors evaluated in this study was good (CV = 14% for PM_2.5_ and 13% for PM_10_). Moreover, the pair‐wise correlation between individual units was very high (*r* > 0.98). The precision of the BB monitors suggests that these monitors respond similarly to organic PM with comparable measurements between units. Our findings are consistent with other studies which demonstrated a high correlation (*r* > 0.9) between measurements obtained from multiple PMS7003 sensors sensor units.^29^, ^32^

Previous calibration reports have used data averaged over hourly intervals to compare monitors incorporating optical PM sensors to the TEOM monitor.^24^, ^29^, ^32^ However, sudden and wide variations in PM concentration occur in indoor^34^ and animal environments.^35^ As monitors developed for assessment of individual exposure must provide sufficient temporal resolution to capture such variation, the difference in response time between optical and TEOM monitors presents a challenge. This study adopted two approaches to address this challenge. First, BB monitors were calibrated with PM data collected during quasi‐steady state conditions. Second, the delayed response of the TEOM monitor was quantified to allow alignment of the data collected from the TEOM and BB monitors.

Variations in temperature and relative humidity were not incorporated into the calibration regression models, though these variables have been shown to affect the performance of the PMS7003 sensors.^29^, ^32^, ^36^ The study was conducted in a temperature‐controlled barn, and as such, experiments occurred under a tight range of temperatures (21.2°C–23.5°C) and relative humidity (12.7%–23.6%). However, the BB monitor logs both temperature and relative humidity every 5 s, which will allow the inclusion of these variables in the analysis of any future exposure studies performed in the field.

Using the BB monitors in a cross‐over feed trial, we demonstrated that soaking hay resulted in a two‐ to three‐fold reduction in exposure to both PM_2.5_ and PM_10_, in agreement with the previous report that breathing zone measures of respirable dust exposure obtained from a single pony were more than halved by completely immersing hay before feeding.^16^ Respirable dust (nominally <4 μm) and PM_2.5_ are closely related,^4^ and measures of PM_2.5_ have been found to provide close approximation of respirable dust in agricultural settings.^37^ In the current study, the extended measure of mean PM_2.5_ exposure when horses were fed soaked hay was higher at 31 μg/m^3^ than the single report for respirable dust at 17 μg/m^3^.^16^ This difference may be explained by differences in hay quality, horse behaviour, and differences in methodology. While the previous report demonstrated good agreement between real‐time DustTrak measures and gravimetric respirable dust measurements in an unoccupied stall, no comparison was made under the higher dust concentrations encountered in the breathing zone of the horse when eating hay.^38^ In the current study, extended measures indicated a mean PM_2.5_ exposure of 76 μg/m^3^ for horses eating dry hay, in general agreement with previously reported gravimetric measures of respirable dust exposure, which range from 55 to 90 μg/m^3^.^5^, ^6^

Previously reported breathing zone measures of PM_10_ exposures range from 72 to 75 μg/m^3^.^30^ Not only are these measures orders of magnitude lower than those reported in this study (1581 μg/m^3^ when horses were fed dry hay), but also they are lower than reported gravimetric measures of respirable dust,^5^, ^30^ though the PM_10_ fraction includes particulates with aerodynamic diameters up to 10 μm. This disagreement among measurements likely reflects the underestimation of PM mass concentration by instruments calibrated with non‐organic particulate matter, Arizona road dust in the case of the DustTrak monitor, and further serves to emphasise the importance of calibration of real‐time monitors with PM pertinent to the environment under investigation. This is also the rationale behind omitting analysis of PM_1_, though this size fraction is reported by the BB monitors. Based upon the large correction factors obtained for PM_2.5_ and PM_10_ during calibration, reporting BB estimates of PM_1_ prior to appropriate calibration of this measure would be inappropriate and potentially misleading. During the cross‐over feed trial, horses were not randomised to feeding assignment. Rather each horse received dry hay on the first day of exposure assessment and soaked hay on the second. While this is a shortcoming in study design arising from the need to accommodate staff schedules, significant influence of results is unlikely as all horses were well accustomed to the monitors prior to the study and the two days of exposure assessment occurred within the same week in a barn under tight environmental control.

Average PM exposures from the first 20 min that the horse had access to forage were compared with those obtained over the course of 8 h. While the data from the initial 20 min tended to overestimate exposures by a factor of 2, both measurement windows demonstrated the benefits of soaking hay. No interaction was found between duration of measurement and type of hay upon PM measures, indicating that the measurement window did not change the effect of hay treatment upon exposure. In other words, for conventionally managed horses, capturing the first 20 minutes of eating is a good representation of the overall exposure. This conclusion greatly facilitates future field studies, where training schedules and personnel limitations often preclude extended sampling periods. It is important to note that the sampling window was standardised to capture the horses' initial interaction with hay, which is the main source of PM exposure in the conventionally managed horse. This measurement window is unlikely to be suitable if the horses' interaction with forage is modified such as with the use of slow feeders or muzzles.

While no respiratory health measures were obtained in this study, a recent study demonstrates that feeding soaked hay for 6 weeks improves lung function in horses suffering exacerbation of severe asthma.^39^ In light of these findings, our data support that soaking hay provides a suitable method to decrease PM exposure to minimise airway inflammation while demonstrating the value of the BB monitor as a tool to evaluate and compare PM exposures of individual horses to both PM_2.5_ and PM_10_. This report provides simple calibration factors for correction and demonstrates the good inter‐unit agreement necessary for reliable individual exposure assessments.

## FUNDING INFORMATION

This project was supported in part by the Grayson‐Jockey Club Research Foundation and the State of Indiana and the Purdue University College of Veterinary Medicine research account funded by the total wager tax. This study was also supported by the USDA National Institute of Food and Agriculture Hatch project 7000907, USA.

## CONFLICT OF INTEREST STATEMENT

The authors have declared no conflicting interests.

## AUTHOR CONTRIBUTIONS

**Kathleen M. Ivester:** Conceptualization; investigation; funding acquisition; writing – original draft; methodology; validation; visualization; writing – review and editing; formal analysis; data curation. **Ji‐Qin Ni:** Conceptualization; investigation; methodology; validation; writing – review and editing; software; formal analysis; data curation; supervision. **Laurent L. Couetil:** Conceptualization; investigation; funding acquisition; methodology; validation; writing – review and editing; project administration; supervision; resources. **Thomas M. Peters:** Conceptualization; investigation; methodology; validation; writing – review and editing; visualization; software; formal analysis; data curation; supervision. **Marcus Tatum:** Investigation; methodology; writing – review and editing; conceptualization; validation. **Lynn Willems:** Investigation; writing – review and editing; data curation. **Jae Hong Park:** Conceptualization; investigation; methodology; validation; writing – review and editing; formal analysis; supervision.

## DATA INTEGRITY STATEMENT

Kathleen M. Ivester had full access to all the data in the study and takes responsibility for the integrity of the data and the accuracy of data analysis.

## ETHICAL ANIMAL RESEARCH

All procedures were approved by the Purdue University Animal Care and Use Committee (Teaching Protocol IACUC 1304000849).

## INFORMED CONSENT

Not applicable.

### PEER REVIEW

The peer review history for this article is available at https://www.webofscience.com/api/gateway/wos/peer-review/10.1111/evj.14425.

## Supporting information


**Data S1.** Supporting Information.

**Data S2.** Supporting Information.

**Figure S1.** Agitator partially filled with alfalfa hay.

**Figure S2.** Study subject flow chart.

**Figure S3.** Simple linear regression plot of TEOM PM_2.5_ versus averaged BB monitors PM_2.5_. Open circles = individual BB data points. BB, Black Beauty monitor. PM_2.5_, particulate matter with an aerodynamic diameter ≤2.5 μm.

**Figure S4.** Simple linear regression plot of TEOM PM_10_ versus averaged BB monitors PM_10_. PM_10_, particulate matter with an aerodynamic diameter ≤10 μm; BB, Black Beauty monitor.

**Table S1.** Supporting Information.

## Data Availability

The data that support the findings of this study are openly available in Purdue University Research Repository (PURR) at https://purr.purdue.edu/publications/4519/1.

## References

[1] Deaton CM , Deaton L , Jose‐Cunilleras E , Vincent TL , Baird AW , Dacre K , et al. Early onset airway obstruction in response to organic dust in the horse. J Appl Physiol. 1985;2007(102):1071–1077. 10.1152/japplphysiol.00264.2006 17158251

[2] Couetil LL , Cardwell JM , Gerber V , Lavoie JP , Leguillette R , Richard EA . Inflammatory airway disease of horses—revised consensus statement. J Vet Intern Med. 2016;30:503–515. 26806374 10.1111/jvim.13824 PMC4913592

[3] Leclere M , Lavoie‐Lamoureux A , Lavoie J‐P . Heaves, an asthma‐like disease of horses. Respirology. 2011;16:1027–1046. 21824219 10.1111/j.1440-1843.2011.02033.x

[4] Ivester KM , Couetil LL , Zimmerman NJ . Investigating the link between particulate exposure and airway inflammation in the horse. J Vet Intern Med. 2014;28:1653–1665. 25273818 10.1111/jvim.12458 PMC4895611

[5] Ivester KM , Couetil LL , Moore GE . An observational study of environmental exposures, airway cytology, and performance in racing thoroughbreds. J Vet Intern Med. 2018;32:1754–1762. 30222207 10.1111/jvim.15226 PMC6189343

[6] Ivester KM , Couetil LL , Moore GE , Zimmerman NJ , Raskin RE . Environmental exposures and airway inflammation in young thoroughbred horses. J Vet Intern Med. 2014;28:918–924. 24773603 10.1111/jvim.12333 PMC4895475

[7] Millerick‐May ML , Karmaus W , Derksen FJ , Berthold B , Holcombe SJ , Robinson NE . Local airborne particulate concentration is associated with visible tracheal mucus in thoroughbred racehorses. Equine Vet J. 2013;45:85–90. 22494157 10.1111/j.2042-3306.2012.00568.x

[8] Millerick‐May ML , Karmaus W , Derksen FJ , Berthold B , Robinson NE . Airborne particulates (pm10) and tracheal mucus: a case‐control study at an american thoroughbred racetrack. Equine Vet J. 2015;47:410–414. 24905487 10.1111/evj.12303

[9] McGorum BC , Ellison J , Cullen RT . Total and respirable airborne dust endotoxin concentrations in three equine management systems. Equine Vet J. 1998;30:430–434. 9758102 10.1111/j.2042-3306.1998.tb04514.x

[10] Olave CJ , Ivester KM , Couetil LL , Franco‐Marmolejo J , Mukhopadhyay A , Robinson JP , et al. Effects of forages, dust exposure and proresolving lipids on airway inflammation in horses. Am J Vet Res. 2021;83:153–161. 34843444 10.2460/ajvr.21.08.0126

[11] Olave CJ , Ivester KM , Couetil LL , Kritchevsky JE , Tinkler SH , Mukhopadhyay A . Dust exposure and pulmonary inflammation in standardbred racehorses fed dry hay or haylage: a pilot study. Vet J. 2021;271:105654. 33840486 10.1016/j.tvjl.2021.105654

[12] Pirie RS , McLachlan G , McGorum BC . Evaluation of nebulised hay dust suspensions (hds) for the diagnosis and investigation of heaves. 1: Preparation and composition of hds. Equine Vet J. 2002;34:332–336. 12117103 10.2746/042516402776249092

[13] Glatter M , Bochnia M , Wensch‐Dorendorf M , Greef JM , Zeyner A . Feed intake parameters of horses fed soaked or steamed hay and hygienic quality of hay stored following treatment. Animals. 2021;11:2729. 34573695 10.3390/ani11092729 PMC8471832

[14] Moore‐Colyer MJ , Taylor JL , James R . The effect of steaming and soaking on the respirable particle, bacteria, mould, and nutrient content in hay for horses. J Equine Vet Sci. 2016;39:62–68.

[15] Moore‐Colyer MJS , Lumbis K , Longland A , Harris P . The effect of five different wetting treatments on the nutrient content and microbial concentration in hay for horses. PLoS One. 2014;9:e114079. 25426729 10.1371/journal.pone.0114079 PMC4245254

[16] Clements JM , Pirie RS . Respirable dust concentrations in equine stables. Part 2: the benefits of soaking hay and optimising the environment in a neighbouring stable. Res Vet Sci. 2007;83:263–268. 17467753 10.1016/j.rvsc.2006.12.003

[17] Ivester KM , Smith K , Moore GE , Zimmerman NJ , Couetilt LL . Variability in particulate concentrations in a horse training barn over time. Equine Vet J. 2012;44(S43):51–56. 23447878 10.1111/j.2042-3306.2012.00647.x

[18] Sousan S , Koehler K , Hallett L , Peters TM . Evaluation of the alphasense optical particle counter (opc‐n2) and the grimm portable aerosol spectrometer (pas‐1.108). Aerosol Sci Tech. 2016;50:1352–1365. 10.1080/02786826.2016.1232859 PMC5580936 28871213

[19] Sousan S , Koehler K , Thomas G , Park JH , Hillman M , Halterman A , et al. Inter‐comparison of low‐cost sensors for measuring the mass concentration of occupational aerosols. Aerosol Sci Tech. 2016;50:462–473. 10.1080/02786826.2016.1162901 PMC5580827 28867868

[20] Castell N , Dauge FR , Schneider P , Vogt M , Lerner U , Fishbain B , et al. Can commercial low‐cost sensor platforms contribute to air quality monitoring and exposure estimates? Environ Int. 2017;99:293–302. 28038970 10.1016/j.envint.2016.12.007

[21] Vercellino RJ , Sleeth DK , Handy RG , Min KT , Collingwood SC . Laboratory evaluation of a low‐cost, real‐time, aerosol multi‐sensor. J Occup Environ Hyg. 2018;15:559–567. 29683781 10.1080/15459624.2018.1468565 PMC6309410

[22] Wang Y , Li J , Jing H , Zhang Q , Jiang J , Biswas P . Laboratory evaluation and calibration of three low‐cost particle sensors for particulate matter measurement. Aerosol Sci Tech. 2015;49:1063–1077.

[23] Sousan S , Koehler K , Hallett L , Peters TM . Evaluation of consumer monitors to measure particulate matter. J Aerosol Sci. 2017;107:123–133. 28871212 10.1016/j.jaerosci.2017.02.013 PMC5580935

[24] Bulot FMJ , Johnston SJ , Basford PJ , Easton NHC , Apetroaie‐Cristea M , Foster GL , et al. Long‐term field comparison of multiple low‐cost particulate matter sensors in an outdoor urban environment. Sci Rep. 2019;9:7497. 31097728 10.1038/s41598-019-43716-3 PMC6522472

[25] Giordano MR , Malings C , Pandis SN , Presto AA , McNeill VF , Westervelt DM , et al. From low‐cost sensors to high‐quality data: a summary of challenges and best practices for effectively calibrating low‐cost particulate matter mass sensors. J Aerosol Sci. 2021;158:105833.

[26] Gramsch E , Oyola P , Reyes F , Vásquez Y , Rubio MA , Soto C , et al. Influence of particle composition and size on the accuracy of low cost pm sensors: findings from field campaigns. Front Environ Sci. 2021;9:751267.

[27] USEPA . List of designated reference and equivalent methods. Vol 75. Research Triangle Park, NC: Office of Research and Development; 2022.

[28] Ni J‐Q , Liu S , Diehl CA , Lim T‐T , Bogan BW , Chen L , et al. Emission factors and characteristics of ammonia, hydrogen sulfide, carbon dioxide, and particulate matter at two high‐rise layer hen houses. Atmos Environ. 2017;154:260–273.

[29] Badura M , Batog P , Drzeniecka‐Osiadacz A , Modzel P . Regression methods in the calibration of low‐cost sensors for ambient particulate matter measurements. SN Appl Sci. 2019;1:622.

[30] Olave CJ , Ivester KM , Couetil LL , Burgess J , Park JH , Mukhopadhyay A . Effects of low‐dust forages on dust exposure, airway cytology, and plasma omega‐3 concentrations in thoroughbred racehorses: a randomized clinical trial. J Vet Intern Med. 2023;37:338–348. 36478588 10.1111/jvim.16598 PMC9889630

[31] Duvall R , Clements A , Hagler G , Kamal A , Kilaru V , Goodman L , et al. Performance testing protocols, metrics, and target values for fine particulate matter air sensors: use in ambient, outdoor, fixed site, non‐regulatory supplemental and informational monitoring applications. Washington, DC: U.S. Environmental Protection Agency, Office of Research and Development; 2021.

[32] Jiang Y , Zhu X , Chen C , Ge Y , Wang W , Zhao Z , et al. On‐field test and data calibration of a low‐cost sensor for fine particles exposure assessment. Ecotoxicol Environ Saf. 2021;211:111958. 33503545 10.1016/j.ecoenv.2021.111958

[33] Sousan S , Regmi S , Park YM . Laboratory evaluation of low‐cost optical particle counters for environmental and occupational exposures. Sensors. 2021;21:4146. 34204182 10.3390/s21124146 PMC8233711

[34] Ciuzas D , Prasauskas T , Krugly E , Sidaraviciute R , Jurelionis A , Seduikyte L , et al. Characterization of indoor aerosol temporal variations for the real‐time management of indoor air quality. Atmos Environ. 2015;118:107–117.

[35] Ni JQ , Erasmus MA , Croney CC , Li C , Li Y . A critical review of advancement in scientific research on food animal welfare‐related air pollution. J Hazard Mater. 2021;408:124468. 33218910 10.1016/j.jhazmat.2020.124468

[36] Levy Zamora M , Xiong F , Gentner D , Kerkez B , Kohrman‐Glaser J , Koehler K . Field and laboratory evaluations of the low‐cost plantower particulate matter sensor. Environ Sci Technol. 2019;53:838–849. 30563344 10.1021/acs.est.8b05174

[37] Jones S , Anthony TR , Sousan S , Altmaier R , Park JH , Peters TM . Evaluation of a low‐cost aerosol sensor to assess dust concentrations in a swine building. Ann Occup Hyg. 2016;60:597–607. 26944922 10.1093/annhyg/mew009 PMC4879606

[38] Clements JM , Pirie RS . Respirable dust concentrations in equine stables. Part 1: validation of equipment and effect of various management systems. Res Vet Sci. 2007;83:256–262. 17477944 10.1016/j.rvsc.2006.12.002

[39] Westerfeld R , Payette F , Dubuc V , Manguin E , Picotte K , Beauchamp G , et al. Effects of soaked hay on lung function and airway inflammation in horses with severe asthma. J Vet Intern Med. 2023;38:469–476. 37930110 10.1111/jvim.16919 PMC10800199

