# Supplementary material for: A wearable real‐time particulate monitor demonstrates that soaking hay reduces dust exposure
Source: Equine Vet J. 2024 Oct 27;57(4):1065–73. doi: 10.1111/evj.14425 (PMC12135757; doi:10.1111/evj.14425)
Supplement: Supplementary file 1 — Data S1. Supporting Information. [file EVJ-57-1065-s001.pdf]

Equine Veterinary Journal BEVA

Data S1:

Particulate Matter Monitors and Optical Particle Sizer:

The Tapered Element Oscillating Microbalance (TEOM) Ambient Particulate Monitor Model 1400a:

The Tapered Element Oscillating Microbalance (TEOM) Ambient Particulate Monitor Model 1400a (Thermo Fisher Scientific, MA, USA) is a U.S. EPA-designated automated equivalent method for PM_{10} and $PM_{2.5}$.[29] Two TEOM monitors, one equipped with a PM_{10} sampling inlet and another equipped with a $PM_{2.5}$ sampling inlet, were used as the reference instruments in the study. The sensor unit outfitted with a PM_{10} sampling inlet was raised 8 cm to place the inlet at the same height as the $PM_{2.5}$ inlet of the other unit. The TEOM filter cartridge in each sensor unit was changed 4-36 hours prior to all tests to ensure optimal responsivity.

The TEOM control units were placed in an antechamber and connected to an on-site computer system (OSCS), which included a personal computer, custom software AirDAC, and data acquisition and control hardware as previously described. [30] The TEOM control units were placed in an antechamber and connected to an on-site computer system (OSCS), which included a personal computer, custom software AirDAC, and data acquisition and control hardware as previously described. [30] The TEOM analog signal outputs were connected to a USB-1680FS (Measurement Computing Co., Norton, MA, USA), from which AirDAC acquired the signals at 1 Hz, converted them to engineering units, and averaged them over 5-second and 60-second intervals. The averaged values were recorded into two data files generated every 24 hours with continual measurement of PM_{10} and $PM_{2.5}$.

The Dylos DC1700-PM $PM_{2.5}$/PM_{10} Air Quality Monitors

The DC1700-PM is a laser particle counter and responds to changes in particle concentration within 6 seconds according to the product manual. Dylos PM measurement data are internally logged as the average of 60-second intervals. The model DC1700-PM outputs PM concentrations in $\mu g/m^3$.
