# Supplementary material for: A wearable real‐time particulate monitor demonstrates that soaking hay reduces dust exposure
Source: Equine Vet J. 2024 Oct 27;57(4):1065–73. doi: 10.1111/evj.14425 (PMC12135757; doi:10.1111/evj.14425)
Supplement: Supplementary file 2 — Data S2. Supporting Information. [file EVJ-57-1065-s003.pdf]

Equine Veterinary Journal BEVA

Data S2:

Test Chamber

Using transparent plastic sheeting and a light wooden frame, the stall (3.2 × 4.1 m^2) was divided into a test chamber (3.2 × 2.1 m^2) with a ceiling height of 2.1 m and an antechamber (3.2 × 2.0 m^2). The test chamber housed the PM monitors, agitation device, and a circulating fan, while the antechamber housed the TEOM control units and computer.

Dylos and BB monitors were suspended from the ceiling frame of the test chamber to ensure that all inlets were at the same height and monitors were evenly spaced 10 cm from the perimeter of the TEOM inlets. The agitator was placed at the corner of the test chamber to facilitate the placement of hay into the agitation chamber and adjustment of agitation speed. By varying the amount of hay in the agitation chamber and the rotation speed of the agitator, quasi-steady state conditions were created over a range of PM concentrations. A box fan was placed in the opposite corner to provide air circulation (Figure 2). Prior to tests, hay was stored in an adjacent horse stall.
