# Supplementary figures and images for: A wearable real‐time particulate monitor demonstrates that soaking hay reduces dust exposure
Source: Equine Vet J. 2024 Oct 27;57(4):1065–73. doi: 10.1111/evj.14425 (PMC12135757; doi:10.1111/evj.14425)

**Figure S1.** Agitator partially filled with alfalfa hay.

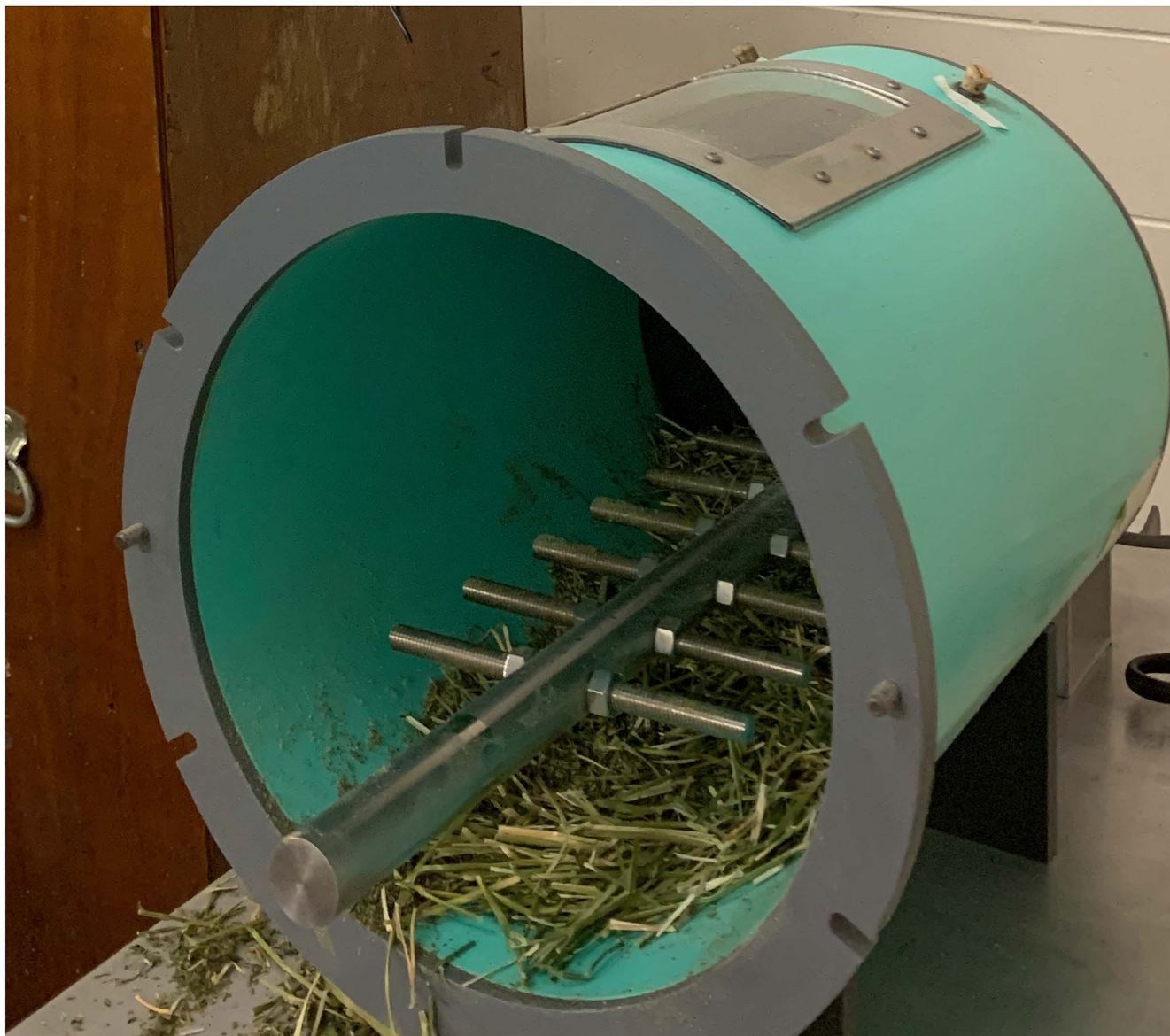

Supplement: Supplementary file 3 — Figure S1. Agitator partially filled with alfalfa hay. [file EVJ-57-1065-s004.pdf]

**Figure S2.** Study subject flow chart.

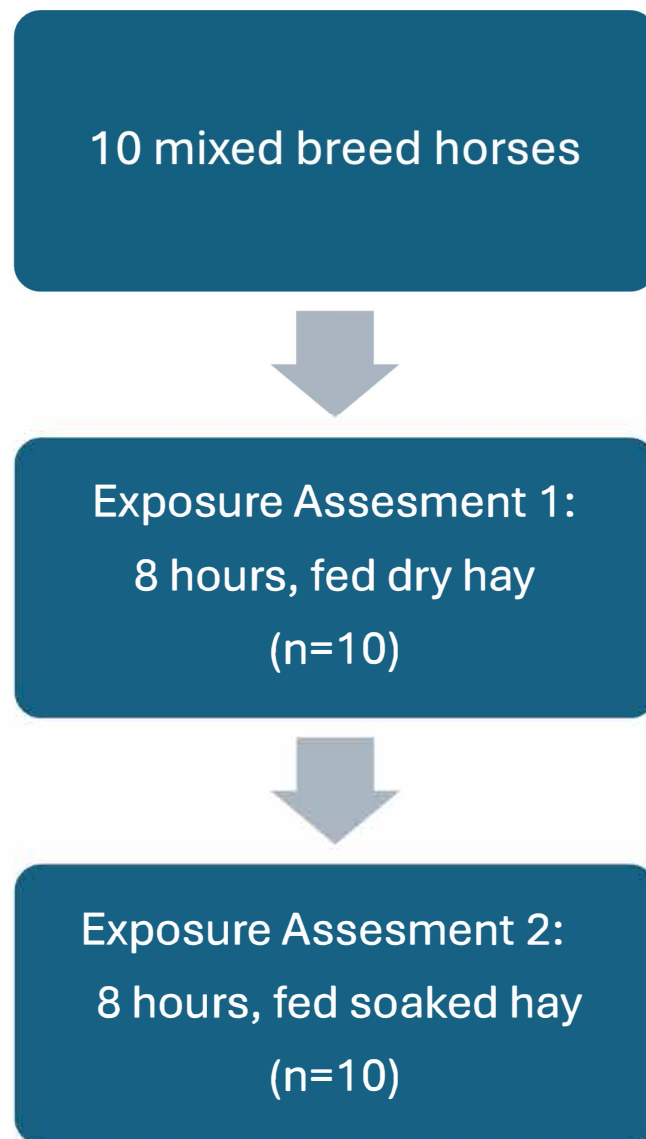

Supplement: Supplementary file 4 — Figure S2. Study subject flow chart. [file EVJ-57-1065-s007.pdf]
