# Supplementary material for: A wearable real‐time particulate monitor demonstrates that soaking hay reduces dust exposure
Source: Equine Vet J. 2024 Oct 27;57(4):1065–73. doi: 10.1111/evj.14425 (PMC12135757; doi:10.1111/evj.14425)

Equine Veterinary Journal BEVA

Figure S3. Simple linear regression plot of TEOM $PM_{2.5}$ vs averaged BB monitors $PM_{2.5}$. Open circles=individual BB data points. Abbreviations: BB, Black Beauty monitor. $PM_{2.5}$, particulate matter with an aerodynamic diameter ≤2.5μm.

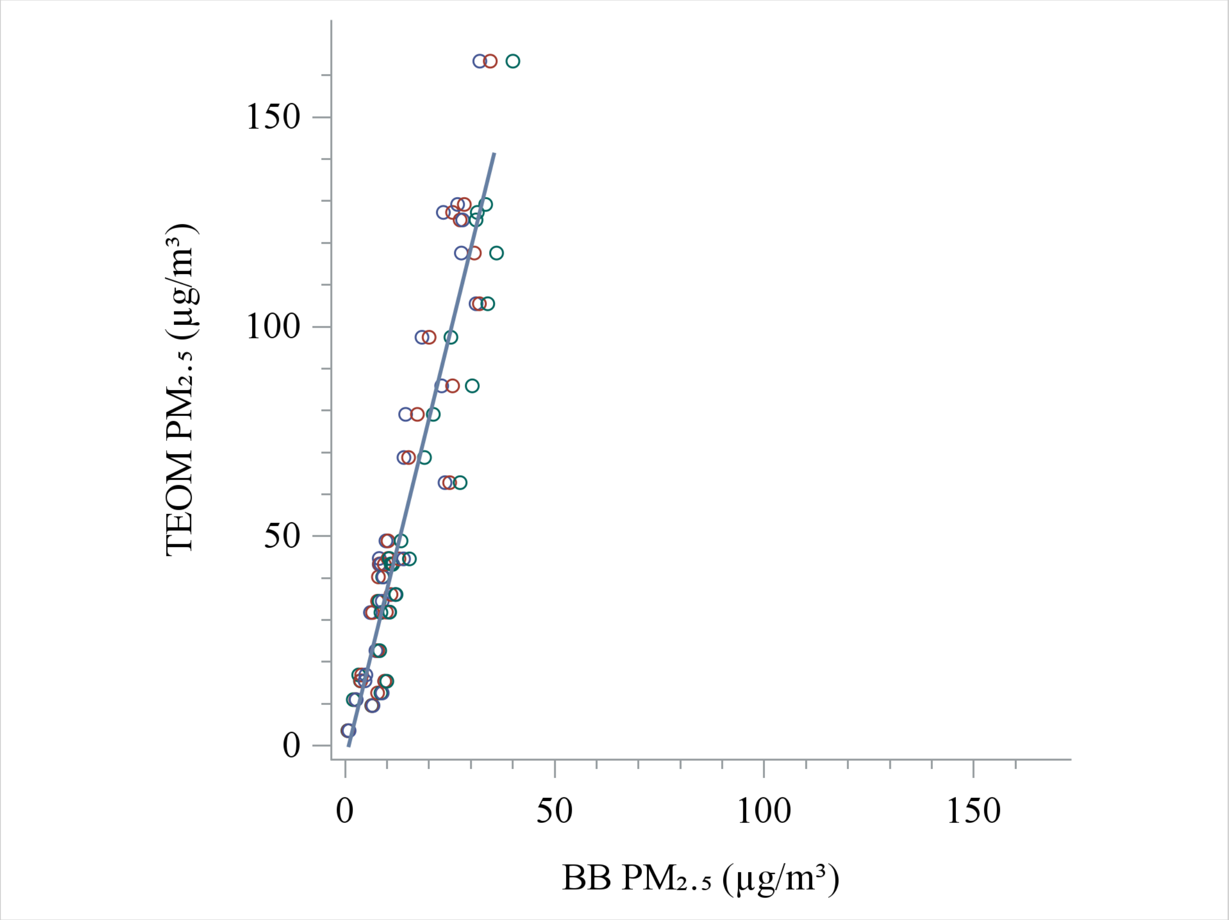

Supplement: Supplementary file 5 — Figure S3. Simple linear regression plot of TEOM PM2.5 versus averaged BB monitors PM2.5. Open circles = individual BB data points. BB, Black Beauty monitor. PM2.5, particulate matter with an aerodynamic diameter ≤2.5 μm. [file EVJ-57-1065-s005.pdf]
