# Supplementary material for: A wearable real‐time particulate monitor demonstrates that soaking hay reduces dust exposure
Source: Equine Vet J. 2024 Oct 27;57(4):1065–73. doi: 10.1111/evj.14425 (PMC12135757; doi:10.1111/evj.14425)

Equine Veterinary Journal BEVA

Figure S4. Simple linear regression plot of TEOM PM_{10} vs averaged BB monitors PM_{10}. PM_{10}, particulate matter with an aerodynamic diameter≤10µm; Abbreviation: BB, Black Beauty monitor.

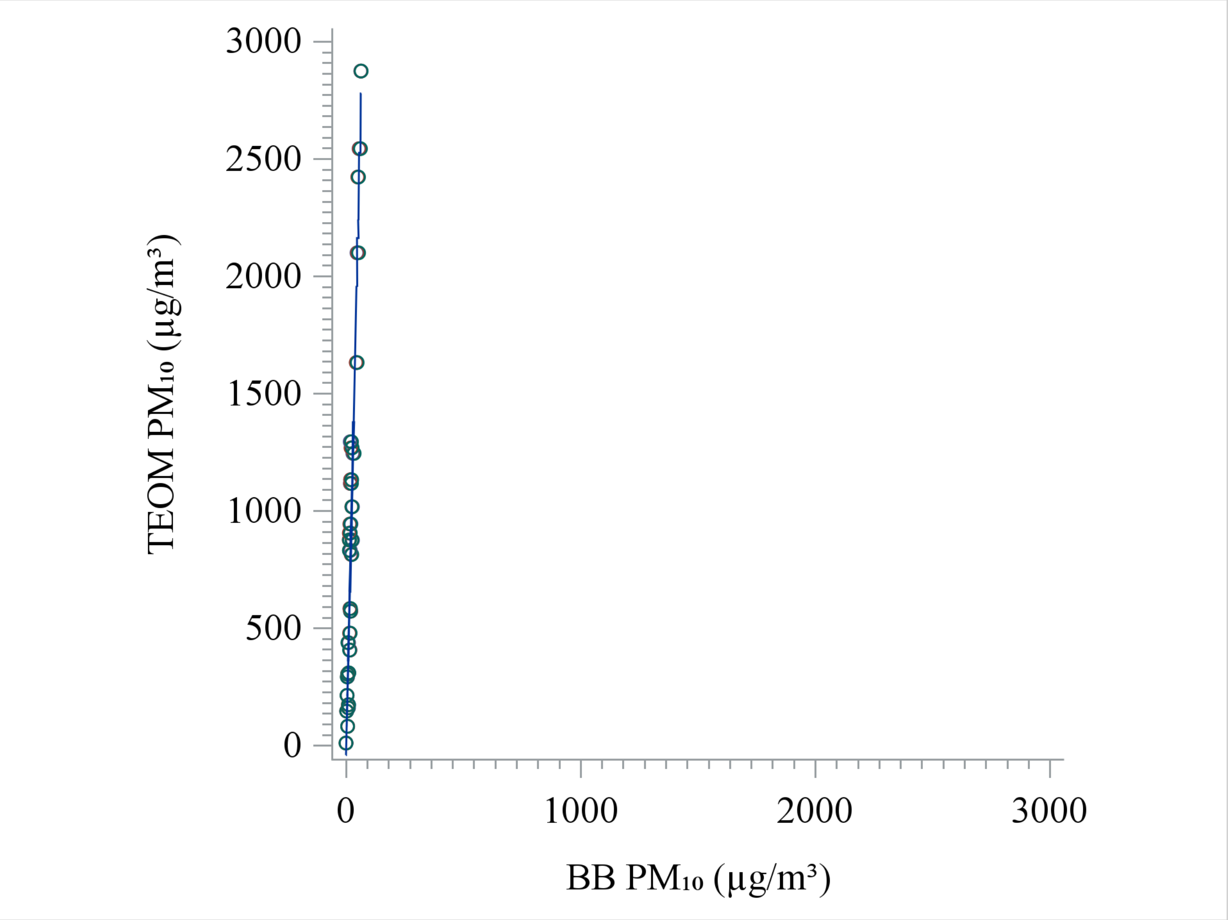

Supplement: Supplementary file 6 — Figure S4. Simple linear regression plot of TEOM PM10 versus averaged BB monitors PM10. PM10, particulate matter with an aerodynamic diameter ≤10 μm; BB, Black Beauty monitor. [file EVJ-57-1065-s002.pdf]
