# Supplementary material for: A wearable real‐time particulate monitor demonstrates that soaking hay reduces dust exposure
Source: Equine Vet J. 2024 Oct 27;57(4):1065–73. doi: 10.1111/evj.14425 (PMC12135757; doi:10.1111/evj.14425)
Supplement: Supplementary file 7 — Table S1. Supporting Information. [file EVJ-57-1065-s006.pdf]

Equine Veterinary Journal BEVA

Table S1: Particulate matter exposure estimates comparing dry hay to soaked hay using short and extended measurements.

Measurement Duration		Dry Hay Mean (95% Confidence Interval)	Soaked Hay Mean (95% Confidence Interval)	p-value
Short	$PM_{2.5}$ (µg/m^3)	160 (110-234)	53.3 (36.5-77.9)	<0.0001
	PM_{10} (µg/m^3)	2829 (2037-3929)	970 (698-1347)	<0.0001
Extended	$PM_{2.5}$ (µg/m^3)	76 (48.4-119)	31 (19.5-47.8)	=0.0008
	PM_{10} (µg/m^3)	1581 (883-2832)	488 (272-874)	=0.0079

Abbreviations: Short, exposure measures obtained for the first 20 minutes after forage was supplied; Extended, exposure measures obtained over the course of 8 hours after forage was supplied; $PM_{2.5}$, particulate matter with an aerodynamic diameter ≤2.5 µm; PM_{10}, particulate matter with an aerodynamic diameter ≤10 µm
